# Exploring the Potential Impact of Creatine Supplementation on Anesthetic Outcomes: A Comprehensive Review

**DOI:** 10.7759/cureus.90661

**Published:** 2025-08-21

**Authors:** Yazen Lehmeidi, Theodoro Beck Neto

**Affiliations:** 1 Anesthesia and Critical Care, SUNY Downstate College of Medicine, New York, USA; 2 Anesthesiology and Perioperative Medicine, SUNY Downstate College of Medicine, New York, USA

**Keywords:** anesthetic outcomes, atp buffering, creatine supplementation, energy metabolism, ketamine, mitochondrial function, neuroprotection, pharmacodynamics, propofol, sevoflurane

## Abstract

Known for its role in energy metabolism and neuromuscular performance, creatine monohydrate is a widely used supplement among athletes and the expanding population of weightlifters. The basis of its use relies on its role in increasing the amount of available ATP, the energy currency of our bodies. Many commonly used anesthetics cause decreases in ATP levels, raising the question of whether creatine may influence the anesthetic process. Despite its popularity and its direct role in providing cellular energy, the effects of creatine supplementation on anesthesia remain underexplored and not completely understood, emphasizing the need for this literature review.

Athletes, a demographic with high creatine supplementation use, are also at elevated risk for musculoskeletal injuries requiring surgical interventions and general anesthesia. Therefore, understanding how chronic creatine use might influence anesthetic outcomes, such as induction time, anesthetic depth, emergence, and hemodynamic stability, is a relevant question with limited available data. This further highlights the need for increased awareness among anesthesiologists regarding creatine use and calls for prospective studies to determine whether supplementation meaningfully alters anesthetic requirements or outcomes. Understanding this interaction may inform preoperative screening and guide personalized anesthesia approaches in a health-conscious surgical population.

This review was conducted to assess the current state of evidence on how creatine may affect anesthetic depth, recovery profiles, and perioperative physiology. A comprehensive literature search was performed across PubMed, Scopus, and Embase using relevant terms. Studies were screened for relevance, resulting in 25 included articles, primarily preclinical studies. Findings suggest that creatine may influence anesthetic pharmacodynamics by modulating intracellular energy stores, calcium handling, nitric oxide signaling, and synaptic function. Animal models show variable responses to anesthetics such as sevoflurane and propofol following creatine preloading, including altered bispectral index (BIS) scores and delayed emergence. However, human data are lacking, and although current clinical practice generally assesses supplement use during preoperative evaluation, creatine use is rarely specifically asked about or considered when dosing.

## Introduction and background

Introduction

Creatine monohydrate is an extensively used dietary supplement, primarily for its capacity to enhance exercise performance, delay fatigue, and support muscle recovery and hypertrophy. Adenosine triphosphate (ATP) is the primary energy-carrying molecule in cells. Creatine acts as a phosphate donor through phosphocreatine, donating a phosphate group to adenosine diphosphate (ADP) and replenishing ATP in tissues with high energy demands [1]. Therefore, phosphocreatine ultimately acts as an energy reserve during increased demand [2]. The mitochondria, commonly referred to as the cell's powerhouse, house cytochrome c in their intermembrane space, which, when released, can initiate a cascade of events that lead to cell death. Substrates of phosphocreatine have been shown to decrease mitochondrial permeability, enhancing mitochondrial integrity [3]. This is important because decreased mitochondrial permeability prevents the release of cytochrome c. This mechanism has drawn attention to creatine monohydrate not only in sports medicine but also in clinical scenarios involving energy dysregulation and modulation.

Many widely administered anesthetics lead to changes in ATP levels in one way or another. Sevoflurane, a commonly used inhaled anesthetic, leads to a decrease in ATP production [4]. Ketamine, another common anesthetic, leads to ATP depletion in neuronal tissue [5]. Propofol, an intravenous anesthetic, leads to suppressed mitochondrial function [6]. Creatine's role in replenishing ATP stores and decreasing mitochondrial stress highlights its potential clinical relevance, effects on required dosage, and protection against neuronal stress and damage. 

Methodology

A comprehensive literature search was conducted using PubMed, Scopus, and Embase for articles published between 1995 and 2024. Keywords included “creatine supplementation,” “anesthesia,” “nitric oxide,” “ATP buffering,” “brain metabolism,” “neuroprotection,” “creatine loading,” and “pharmacodynamics.” Only English-language research articles and review papers were considered.

Titles and abstracts were first screened for relevance to creatine’s biochemical actions, pharmacokinetics, or its interactions with anesthesia. Full-text articles were then reviewed and prioritized based on several criteria: (1) the relevance of species studied, with a preference for human data and translationally valid animal models such as rats and mice; (2) inclusion of anesthetic-specific endpoints such as minimum alveolar concentration (MAC), bispectral index (BIS), induction time, emergence profile, or postoperative recovery metrics; and (3) demonstration of mechanistic insights related to ATP levels, mitochondrial dynamics, calcium homeostasis, or oxidative stress.

**Figure 1:**
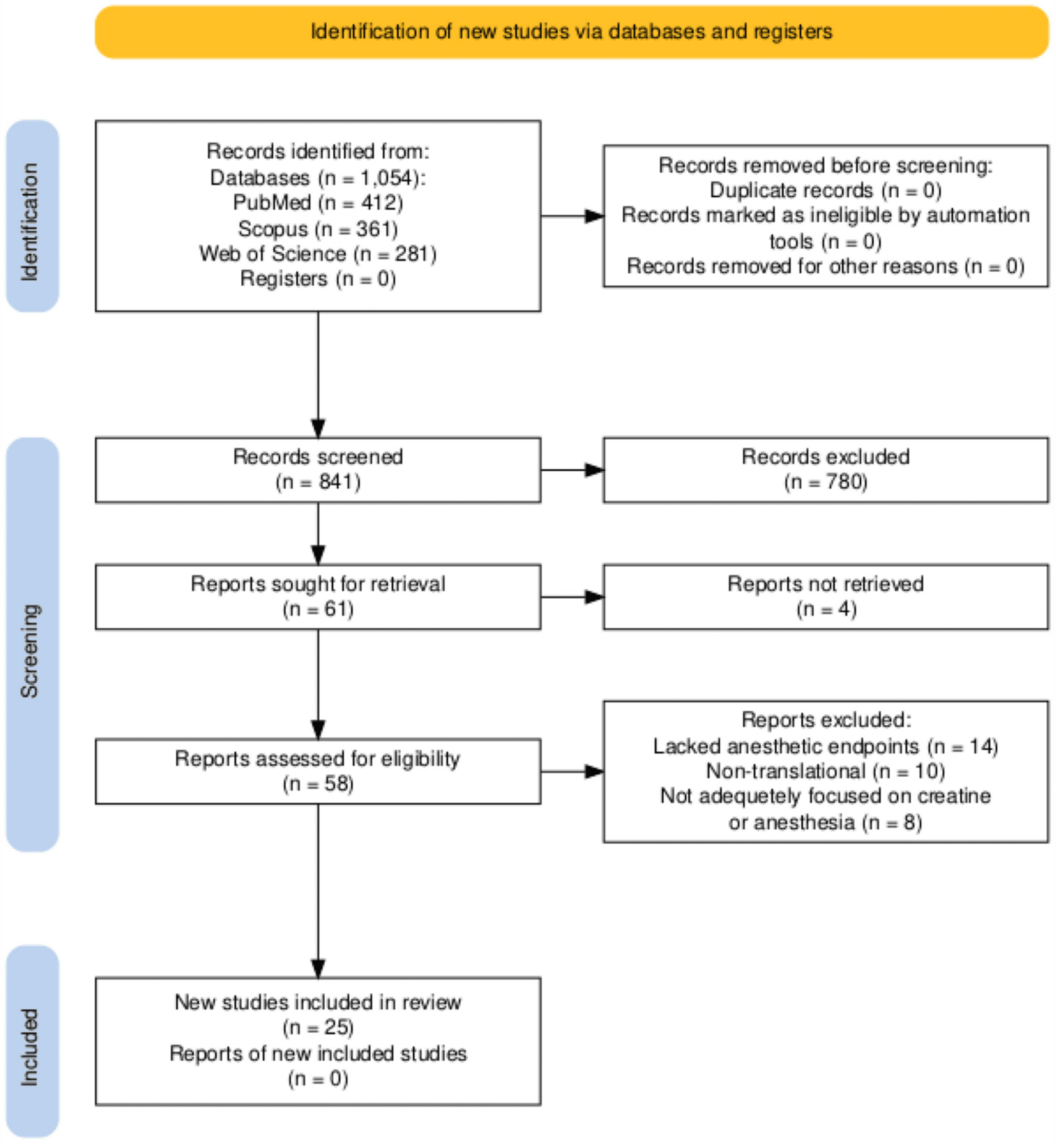
PRISMA 2020 flow diagram illustrating the selection process for studies on creatine and anesthesia, including records identified (n=1054), screened (n=841), and included (n=25).

Studies involving clinically relevant anesthetic agents (e.g., propofol, sevoflurane, and ketamine) and well-defined creatine dosing protocols were given higher priority. Preference was also given to experiments using quantitative physiological or neurophysiological measures and studies offering clear insight into the overlap between creatine bioenergetics and anesthetic pharmacodynamics or neuroprotection. No statistical summary methods are used. Figure 1 is a PRISMA flow diagram illustrating this selection process.

## Review

Biochemical basis of creatine supplementation

Creatine is synthesized endogenously in the liver and kidneys or obtained from dietary sources such as red meats. It is phosphorylated by creatine kinase to form phosphocreatine, a rapidly mobilized reserve of high-energy phosphates that buffers ATP levels by phosphorylating AMP and ADP during increased metabolic demand [2]. Supplementation with creatine monohydrate significantly increases total body creatine stores, particularly in muscle and brain tissues. For example, a typical loading phase (20 g/day for five to seven days) can increase intramuscular creatine content by 10-40% [7,8]. This elevated reservoir enhances ATP buffering capacity, improves calcium handling, and supports mitochondrial function. As a result, these biochemical enhancements may potentially alter the way tissues respond to metabolic suppressors such as anesthetics. Beyond energy metabolism, creatine also supports mitochondrial integrity, regulates calcium homeostasis, and modulates oxidative stress [9,10]. Notably, Engl and Garvert proposed a prophylactic role for creatine in hypoxia-related neurologic injury, which parallels several mechanisms involved in anesthetic suppression [11].

Mechanistic insights into anesthetic agents and energy metabolism

Propofol, a widely used intravenous anesthetic, exerts its effects primarily through potentiation of GABA-A receptors, leading to increased inhibitory neurotransmission and neuronal hyperpolarization [6]. This action reduces cerebral metabolic rate (CMR) and suppresses mitochondrial function. These effects may be influenced by the energy-preserving functions of creatine, particularly in tissues with high metabolic demand. By enhancing mitochondrial resilience and ATP buffering, creatine could theoretically influence the depth of anesthesia required or help maintain neuronal function under propofol-induced suppression.

Similarly, sevoflurane, a volatile inhaled anesthetic, enhances GABAergic transmission while also inhibiting NMDA receptors. It suppresses mitochondrial oxidative phosphorylation and decreases ATP production [4]. Given creatine’s role in preserving ATP levels and preventing mitochondrial collapse, individuals with higher creatine reserves may demonstrate altered anesthetic sensitivity to sevoflurane, potentially requiring adjustments in dose to achieve equivalent sedation or emergence profiles. Ketamine, a dissociative anesthetic commonly used for induction and analgesia, exerts its effects through noncompetitive antagonism of NMDA receptors. This mechanism leads to decreased excitatory neurotransmission but has also been associated with mitochondrial dysfunction, increased oxidative stress, and ATP depletion in neuronal tissue [5]. Given creatine’s role in preserving mitochondrial integrity and buffering cellular energy by increasing ATP stores, supplementation could mitigate some of ketamine’s metabolic stress. These findings suggest that creatine might alter ketamine’s onset or duration by modulating the energetic state of neural circuits.

Anesthesia monitoring parameters: BIS and MAC

The BIS and MAC are commonly used to monitor anesthetic depth and potency. BIS derives a numerical value (0-100) from processed EEG signals, providing an estimate of a patient’s level of consciousness under anesthesia; the lower the value, the deeper the sedation [12]. MAC, on the other hand, refers to the concentration of an inhaled anesthetic in the alveoli required to prevent movement in 50% of subjects in response to a noxious stimulus; it serves as a standard measure of anesthetic potency and is influenced by age, temperature, and comorbidities [13]. Since creatine supports neuronal ATP availability and mitochondrial function, it is plausible that supplementation could delay cortical suppression under anesthesia, resulting in higher BIS values at equivalent drug concentrations. Similarly, creatine’s impact on cerebral energetics could affect MAC by modifying the brain’s responsiveness to volatile agents. These potential shifts emphasize the need for further study into how creatine status may confound anesthetic titration and monitoring.

Mechanistic overlap between creatine and anesthetic pathways

The mechanisms of general anesthesia and the bioenergetic functions of creatine intersect at several important cellular and molecular levels. Anesthetics such as propofol and sevoflurane impair mitochondrial respiration, decrease CMR, and disrupt calcium balance [6,4]. These same domains are supported by creatine and phosphocreatine, especially through their roles in ATP regeneration and buffering during high-energy demand. In particular, creatine enhances mitochondrial integrity via the creatine kinase-phosphocreatine system and supports micro-compartmentalized ATP delivery near sites of consumption. Dolder demonstrated that phosphocreatine substrates inhibit the mitochondrial permeability transition pore, a key regulator of cell death under stress conditions [3]. By stabilizing mitochondrial dynamics, creatine may counteract anesthetic-induced mitochondrial depression. This mitochondrial protection underscores one of the primary biochemical overlaps between creatine and anesthetic agents.

Intracellular calcium homeostasis represents another shared regulatory pathway. Anesthetics are known to disrupt calcium handling, which contributes to impaired neurotransmission and increases the risk of apoptosis. In contrast, creatine helps maintain ATP-dependent calcium pumps and buffering systems [9]. Creatine may, therefore, help preserve calcium regulation even under anesthetic challenge. This potential to stabilize calcium dynamics under anesthesia may be particularly relevant during surgeries involving prolonged exposure to anesthetics. Moreover, oxidative stress is a common consequence of anesthesia, especially in high-risk populations such as neonates and the elderly. Lawler et al. demonstrated that creatine possesses direct antioxidant properties and supports endogenous defense systems [14]. These effects suggest that creatine could help mitigate oxidative neuronal injury during anesthetic exposure.

Further supporting this hypothesis, Kass and Lipton provided early experimental evidence that creatine delays irreversible anoxic injury in hippocampal slices, highlighting its ability to preserve neuronal viability under energy-deprived conditions [15]. Complementing this, Bresnen and Duong demonstrated that increasing isoflurane concentration significantly reduced cerebral creatine kinase metabolic flux by over 40%, suggesting that anesthetics impair energy turnover even when ATP and phosphocreatine concentrations remain stable [16]. The interdependence of these findings implies physiological reciprocity: if anesthesia reduces brain phosphocreatine stores, then creatine supplementation may, in turn, influence anesthetic sensitivity, required dose, or recovery kinetics. This strengthens the argument for studying creatine as a modulator of anesthetic pharmacodynamics. Importantly, this modulatory potential could be especially meaningful in populations where metabolic resilience is already compromised.

Finally, both anesthetics and creatine reduce neuronal excitability and metabolic demand, contributing to neuroprotection in ischemic or hypoxic conditions. However, this overlap may also mean that creatine supplementation could reduce the effective concentration of anesthetics required to achieve cortical suppression. As a result, patients with elevated phosphocreatine levels might recover consciousness more rapidly, demonstrate a lower risk of postoperative cognitive dysfunction, or respond differently to agents titrated to BIS or MAC targets. Taken together, the mechanistic overlap suggests that creatine may not only provide cellular protection but also modulate how anesthetic agents act on the brain and other high-energy demand tissues. This reinforces the need for clinical studies assessing anesthetic requirements and recovery outcomes in creatine-supplemented individuals.

Animal and experimental insights

Several animal studies have helped shed light on how creatine supplementation may interact with anesthesia via energy metabolism, neuroprotection, and oxidative stress regulation. Saberi et al. investigated the impact of creatine and whey protein supplementation on anesthesia in rats [17]. Their study demonstrated changes in both induction time and recovery profile following anesthesia, suggesting that creatine alters anesthetic pharmacodynamics. This can potentially be explained by cellular energy modulation. Venâncio et al. administered ketamine to rats and observed mitochondrial dysfunction, ATP depletion, and increased oxidative stress [5]. While creatine was not administered in this study, the findings reinforce ketamine’s energy-disruptive profile, highlighting a potential therapeutic niche for creatine in buffering these adverse effects. Bresnen and Duong used in vivo phosphorus-31 magnetization transfer spectroscopy to show that isoflurane anesthesia significantly reduced cerebral creatine kinase flux, indicating impaired energy metabolism despite stable ATP and phosphocreatine levels [16]. This provides direct evidence that anesthetics impair brain energy metabolism, an effect that may be mitigated by creatine supplementation.

Supporting this idea, Wheatley and McLoughlin reported decreased ATP levels in both skeletal muscle and brain under anesthesia, reinforcing the concept that general anesthesia induces systemic energetic stress [18]. Because creatine preferentially accumulates in these high-demand tissues, supplementation may offer a physiological buffer. Further compounding this vulnerability, Tétrault et al. demonstrated that isoflurane anesthesia disrupts the blood-brain barrier (BBB) in cats, potentially impairing nutrient transport and increasing neural susceptibility to metabolic stress [19]. Since creatine relies on intact BBB transport mechanisms to enter the brain, such disruption may hinder its cerebral uptake during anesthesia. Therefore, maintaining elevated baseline creatine levels through supplementation could preserve neuroenergetic stability when BBB integrity is compromised.

Together, these studies suggest a convergence between anesthesia-induced energy depletion and creatine’s bioenergetic support. Table 1 summarizes these key findings.

**Table 1:** Summary of key animal findings. ATP, adenosine triphosphate; ^31^P-MT-MRS, phosphorus-31 magnetization transfer magnetic resonance spectroscopy; BBB, blood-brain barrier

Study	Animal model	Intervention	Findings	Relevance
Saberi et al., 2016 [17]	Rats	Creatine ± whey + anesthesia	Altered induction and recovery times	Indicates modulation of anesthetic pharmacodynamics via energy metabolism
Venâncio et al., 2011 [5]	Rats	Ketamine only	Mitochondrial dysfunction, ATP depletion, oxidative stress	Reinforces ketamine’s energy-disruptive effects; creatine may counteract these
Bresnen & Duong, 2015 [16]	Rats	Isoflurane anesthesia	Decreased creatine kinase flux despite stable ATP and phosphocreatine (via ³¹P-MT-MRS)	Demonstrates that isoflurane impairs cerebral energy turnover; creatine may support this system
Wheatley & McLoughlin, 1987 [18]	Rats	General anesthesia	Reduced ATP in brain and skeletal muscle	Supports the systemic energy-depleting effects of anesthesia; creatine targets the same tissues
Tétrault et al., 2008 [19]	Cats	Isoflurane anesthesia	Isoflurane increased BBB permeability	Suggests anesthesia may impair BBB integrity; creatine’s brain uptake could be compromised or protective

Clinical implications for perioperative care

Preoperatively, patients who are regular users of creatine or those considered for supplementation may have altered responses to anesthetics due to increased ATP availability and mitochondrial preservation. This may result in differences in anesthetic depth, induction times, or emergence profiles. While animal studies suggest such interactions, clinical data are lacking. Therefore, anesthesiologists should consider closely monitoring patients on creatine, particularly using tools like MAC and BIS to assess anesthetic depth [1,4]. Additionally, a 2023 study by Hogenbirk et al. found that nearly 40% of patients undergoing major abdominal cancer surgery experienced clinically significant muscle loss, defined as a ≥10% reduction in rectus femoris cross-sectional area, within just one week postoperatively [20]. In a randomized human study, Hespel et al. found that creatine supplementation after immobilization led to significantly faster recovery of quadriceps muscle cross-sectional area (~10% loss reversed) and strength [21]. Together, these studies suggest that creatine supplementation may help prevent or accelerate recovery from muscle loss associated with anesthesia and surgery. Another critical factor is the incidence of hypoxia-induced damage: approximately 40% of critically ill surgical patients develop post-anesthesia hypoxia, which may lead to complications like cognitive dysfunction and muscle weakness [22]. Given its role in mitochondrial function and ATP buffering, creatine has neuroprotective properties that could mitigate some of the neuronal damage associated with hypoxia [10]. Supplementing with creatine might help preserve cellular energy reserves in tissues vulnerable to oxygen deprivation, reducing the incidence of postoperative complications such as cognitive dysfunction and muscle weakness.

Furthermore, elderly individuals represent a particularly vulnerable population with respect to both muscle wasting and cognitive dysfunction following surgery. Studies suggest that creatine supplementation can significantly improve muscle mass, strength, and cognitive function in elderly populations [23,24]. Creatine may also help protect against neurotoxic effects and improve recovery post-anesthesia, as it has been shown to yield cognitive enhancements in the elderly [25]. To achieve these benefits, the use of creatine in perioperative patients may follow a specific dosing regimen: begin with a loading phase of 20 grams/day for five to seven days, divided into four doses of 5 grams each. This phase increases muscle creatine stores by 20-40% [7], providing enhanced ATP buffering capacity and supporting mitochondrial function. After the loading phase, a maintenance dose of 3-5 grams/day can be continued to sustain elevated creatine levels [7]. Kidney function should be closely monitored, especially in patients with preexisting renal conditions, through baseline tests like serum creatinine levels and glomerular filtration rate (GFR), as creatine is metabolized into creatinine and excreted by the kidneys [1]. Ultimately, this regimen can help mitigate postoperative muscle wasting, preserve cognitive function, and offer neuroprotective benefits, particularly in elderly and critically ill patients [21,25].

Limitations and future research directions

Despite promising findings from animal studies, the literature examining creatine’s impact on anesthetic outcomes remains limited, with a lack of robust clinical evidence to guide practice. Most available data stem from preclinical models, leaving a significant gap in understanding how creatine supplementation may influence anesthesia in human populations. To address this, future research should prioritize well-designed randomized controlled trials (RCTs) comparing creatine supplementation to placebo in surgical patients. These studies should include preoperative loading phases and evaluate postoperative outcomes such as muscle strength, cognitive recovery, and anesthetic depth. Target populations should include elderly patients, critically ill individuals, and those undergoing neurosurgery, as these groups are particularly vulnerable to postoperative complications and may stand to benefit the most from creatine’s potential protective effects.

Key outcomes to measure should include both physiological and functional parameters. Primary endpoints could involve MAC values, BIS scores, postoperative cognitive testing, and strength assessments, while secondary outcomes may include incidence of postoperative delirium, hospital length of stay, and complications such as hypoxia or muscle wasting. Rigorous statistical approaches, such as mixed-effects models, are essential to control for repeated measures and confounding variables like baseline comorbidities or concurrent medications. Subgroup analyses should be performed to explore differential effects across high-risk populations. These efforts will be critical in translating promising mechanistic findings into actionable clinical strategies.

## Conclusions

This review highlights a growing body of preclinical evidence suggesting that creatine supplementation may influence anesthetic pharmacodynamics by modulating ATP buffering, mitochondrial stability, and oxidative stress. Animal studies demonstrate that creatine can alter anesthetic responses, including prolonged analgesia, changes in induction and emergence times, and preservation of cerebral energy metabolism during exposure to agents like ketamine, sevoflurane, and propofol. These effects are mechanistically supported by creatine’s known roles in stabilizing mitochondrial function and preserving intracellular ATP levels. However, clinical data remain lacking, and routine anesthetic practice does not directly consider supplement use, despite widespread creatine consumption in populations such as athletes, the elderly, and critically ill patients.

Taken together, this review's findings support a central hypothesis: creatine supplementation may modulate anesthetic pharmacodynamics, thereby altering the required dose for induction, depth of anesthesia, or speed of emergence. These interactions could theoretically impact anesthetic depth through greater neuronal ATP availability or facilitate faster recovery by preserving metabolic resilience. Given this plausible bidirectional effect, future studies should test this hypothesis using controlled human trials that measure anesthetic requirements, BIS and MAC values, and recovery profiles in creatine-loaded versus non-supplemented individuals. Until such data emerges, clinicians should consider screening for creatine use preoperatively and recognize its potential to confound anesthetic monitoring and recovery expectations.
